# Exosomal long non-coding RNAs in gastrointestinal cancer: chemoresistance mediators and therapeutic targets

**DOI:** 10.1186/s12967-025-06878-5

**Published:** 2025-08-08

**Authors:** Chenhe Li, Shunjia Xing, Dechun Zhang, Ruoyu Li, Qian Li, Hongliang Luo, Fangteng Liu

**Affiliations:** 1 Department of Clinical Medicine, School of Queen Mary, Nanchang University, Jiangxi, 330006 People’s Republic of China; 2 Department of Oncology, Shanghai Children’s Medical Center, Shanghai Jiao Tong University School of Medicine, Shanghai, 200127 PR China; 3 Department of Gastrointestinal Surgery, The Second Affiliated Hospital, Jiangxi Medical College, Nanchang University, Nanchang, 330008 Jiangxi China

**Keywords:** Gastrointestinal cancers, Exosomal lncRNAs, Intercellular communication, Chemoresistance, Therapeutic targets

## Abstract

Gastrointestinal (GI) cancer is a series of malignant cancer mainly affecting the GI tract. Chemotherapy is one of the most treatment strategies used in GI cancer treatment, especially in advanced cases. However, the challenge of chemoresistance significantly compromises treatment success, resulting in poorer clinical outcomes and increased metastatic potential. Long non-coding RNAs (lncRNAs) have emerged as important regulators of gene expression and are implicated in various disease processes. Recent research has demonstrated that lncRNAs can be packaged within exosomes, thereby facilitating intercellular communication and potentially transferring chemoresistance traits among cancer cells. This review focuses on the biogenesis and functional roles of exosomal lncRNAs in promoting chemoresistance across different GI cancers. We highlight specific lncRNAs, elucidate their mechanisms of action, and discuss innovative therapeutic strategies aimed at targeting these molecules in gastrointestinal malignancies. By exploring these intricate interactions, we aim to identify novel approaches to overcome chemoresistance and improve the efficacy of treatments for GI cancer.

## Introduction

Gastrointestinal (GI) cancers, including those of the esophagus, stomach, pancreas, colon, hepatic and rectum, are among the most prevalent and deadly malignancies globally. These cancers exhibit diverse pathogenic mechanisms, complicating treatment approaches [1]. Chemotherapy is a cornerstone of treatment, particularly for advanced GI cancers, where metastasis or surgical limitations necessitate systemic interventions. A critical challenge in the treatment of GI cancers is the development of chemoresistance, where cancer cells become unresponsive to chemotherapy. This reduces treatment efficacy and worsens patient outcomes [2]. Chemoresistance often leads to the need for alternative therapies or higher drug doses, increasing toxicity. It can be intrinsic or acquired, driven by various factors such as drug efflux mechanisms, altered drug targets, enhanced DNA repair, or apoptosis evasion. Genetic and epigenetic changes, as well as the tumor microenvironment, play key roles in this resistance [3].

Long non-coding RNAs (lncRNAs), RNA molecules over 200 nucleotides long, have emerged as important regulators in cancer biology. Although they are traditionally recognized as not coding for proteins, lncRNAs influence gene expression at multiple levels, including epigenetic, transcriptional, and post-transcriptional regulation through several different pathways [4–6]. Exosomes, which are lipid bilayer vesicles secreted into the extracellular space, have gained attention for their role in transporting bioactive molecules, such as proteins and RNAs, between cells [7, 8]. It exists in a variety of biological fluids, such as serum, plasma, urine, saliva, ascites, cerebrospinal fluid, and amniotic fluid. These vesicles can influence tumor progression, metastasis, and drug resistance [9–11].

In GI cancers, various exosomal lncRNAs have been identified as mediators of chemoresistance across different cancer types [12–14]. For instance, exosomal lncRNA H19 is associated with drug resistance in colorectal cancer [15], while exosomal lncRNA PVT1 is implicated in gastric cancer [16]. The transfer of lncRNAs through exosomes facilitates intercellular communication, mediating tumor survival and resistance to treatment. Due to their stability and tissue specificity, exosomal lncRNAs hold great promise as biomarkers for liquid biopsies and as potential therapeutic targets [17].

This review synthesizes current findings on the role of exosomal lncRNAs in chemoresistance in GI cancers, highlighting specific exosomal lncRNAs and their potential as therapeutic targets to regulate chemotherapy sensitivity in these malignancies.

## Exosomal lncRNAs: introduction and function

### Formation and release of exosomal lncRNAs

Exosomes, extracellular vesicles ranging from 30 to 150 nm in size, are secreted by a variety of mammalian cells. Structural analysis reveals that exosomes possess a lipid bilayer membrane and lack cellular organelles. Their surface components vary depending on the source cells and tissues [18], yet most exosomes contain evolutionarily conserved proteins and lipids, such as glycosylated proteoglycans, tetraspanins (including CD9, CD63, and CD81), and heat shock proteins like HSP60 and HSP905-7 [19]. Exosome biogenesis begins with the invagination of the plasma membrane, leading to the formation of early endosomes. These early endosomes mature into late endosomes or multivesicular bodies, which contain intraluminal vesicles (ILVs) [3, 20].

**Fig. 1:**
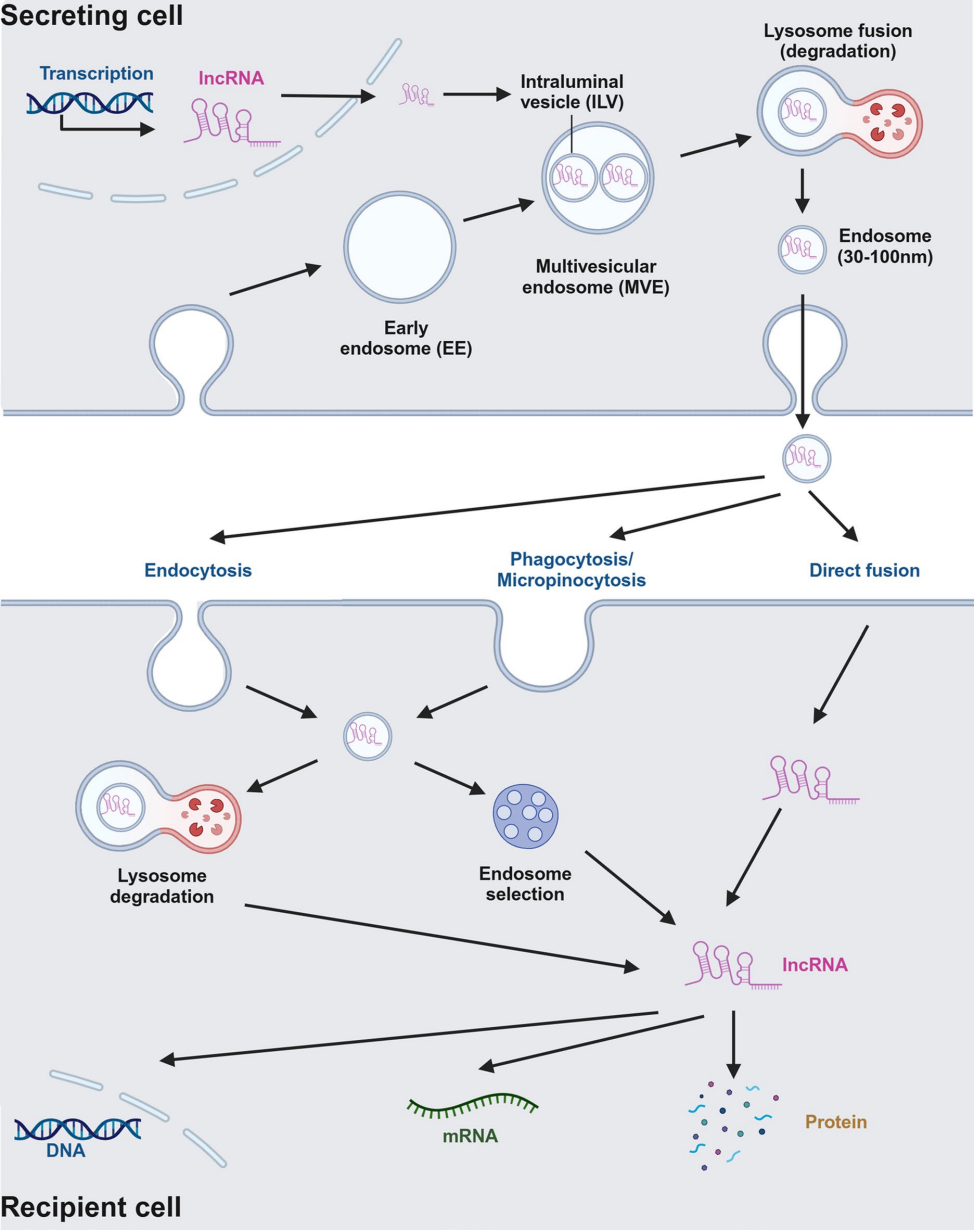
Mechanisms of Exosome-Mediated lncRNA Transfer between Secreting and Recipient Cells. This figure illustrates the detailed mechanisms by which long non-coding RNAs (lncRNAs) are packaged, secreted, and transferred between cells via exosomes. Exosomes, small extracellular vesicles (30–100 nm), originate from intraluminal vesicles (ILVs) within multivesicular endosomes (MVEs) in the secreting cell. The ESCRT complex facilitates the formation of MVEs, which either fuse with lysosomes for degradation or with the plasma membrane to release exosomes. Once exosomes enter the extracellular space, they can be taken up by recipient cells through various processes, including phagocytosis, micropinocytosis, endocytosis, or direct fusion with the cell membrane. Upon entry into the recipient cell, exosomes are either directed towards lysosomal degradation or processed through the endosomal pathway. The lncRNAs carried within these exosomes can then be released and act on the recipient cell's gene regulatory machinery, affecting mRNA translation and protein production, and modulating diverse cellular functions. This mechanism highlights the significance of exosome-mediated lncRNA transfer in intercellular communication and gene regulation. Created in BioRender

The endosomal sorting complex required for transport (ESCRT) and tetraspanins play critical roles in sorting and loading cargo into ILVs [21]. The MVBs may then either fuse with lysosomes for degradation or with the plasma membrane to release ILVs as exosomes into the extracellular space [4, 22]. This process is influenced by several factors, including the presence of specific proteins and lipids. For instance, neutral sphingomyelinase-2 and Rab27a enhance exosome production by activating the nSMase and Rab GTPase pathways [23]. Once exosomes are released into the extracellular environment, they can be picked up by other cells through several pathways, including endocytosis, direct fusion, phagocytosis and micropinocytosis [24]. In summary, exosomes originate from the endosomal system and are released into the extracellular environment when MVBs fuse with the plasma membrane. Extracellular exosomes then are picked up by cells through different pathways (Fig. 1).

Exosomes play a pivotal role in intercellular material transfer and signal transduction. They transport biologically active molecules, such as mRNAs, miRNAs, proteins, and lipids, to recipient cells, thereby enabling cell-to-cell communication, providing target cells with new surface molecules, and facilitating the exchange of membrane proteins and cytoplasmic material between cell types. Moreover, exosomes contribute to the removal of unnecessary membrane proteins, such as transferrin receptors, functioning similarly to lysosomes [25]. Exosomes also play a key role in the immune system, where exosomes derived from antigen-presenting cells can stimulate major histocompatibility complex molecule antigen presentation, mimicking B-cell behavior [26]. Phase I clinical trials have demonstrated that dendritic cell-derived exosomes, carrying tumor peptides, can induce T-cell-mediated anti-tumor immune responses, which in some instances are more effective than dendritic cells themselves [27]. These biological functions of exosomes lay the groundwork for the transport of lncRNAs and the regulation of tumor resistance.

Specific RNA-binding proteins, such as HNRNPA2B1 and ALIX, facilitate the selective packaging of lncRNAs into exosomes for transport between cells [28]. Encapsulation within exosomes not only enables the long-distance transmission of lncRNAs across different cells, tissues, and organs but also protects lncRNAs from degradation by extracellular ribonucleases [29]. Exosomal lncRNAs, therefore, combine the long-range signaling capabilities of exosomes with the regulatory potential of lncRNAs, making them central players in modulating the tumor microenvironment. By regulating gene expression, signaling pathways, and drug efflux, exosomal lncRNAs promote the spread of chemoresistance in cancer and serve as crucial modulators within the oncogenic network [30].

### The role of exosomal lncRNA in tumor drug resistance

The pivotal role of exosomal lncRNAs in mediating cancer chemoresistance has garnered increasing scrutiny. Exosomes emerge as crucial mediators of intercellular communication, facilitating the transfer of biomolecules—particularly lncRNA. Evidence indicates that these lncRNAs regulate cancer drug resistance through diverse pathways, existing within a complex network of biological interactions. This intricate web encompasses gene interactions, gene and microRNA (miRNA) dynamics, protein–protein associations, parallel signaling cascades, and various forms of cellular communication. The dynamic interplay within this network hinges on the continuous exchange of biological substances across nuclear and plasma membranes [18].

Specifically, exosomal lncRNAs can modify cellular behaviors within the tumor microenvironment by transmitting critical genetic information. For example, exosomes from gastric cancer cells can induce widespread alterations in lncRNA expression in mesenchymal stem cells, influencing their differentiation and promoting tumor progression [31]. Similarly, exosomes derived from glioblastoma multiforme cancer stem cells can transform the phenotype of parenchymal cells, facilitating tumor growth and immune evasion [32].

Under hypoxic conditions, tumor cells upregulate the biogenesis and secretion of exosomes, thereby enhancing metastasis and angiogenesis by modifying exosomal cargo. These mechanisms affirm the vital role of exosomal lncRNAs in orchestrating the tumor microenvironment, particularly regarding drug resistance and invasiveness [33]. For instance, exosomes from CD90 + liver cancer cells contain lncRNA H19, which can be internalized by endothelial cells, promoting an angiogenic phenotype and enhancing intercellular adhesion [34]. This emphasizes the regulatory capacity of exosomal lncRNAs in tumor-associated angiogenesis.

Overexpression of ATP-binding cassette (ABC) transporters (such as ABCB1 and ABCG2) in cancer cells is one of the main mechanisms of multidrug resistance. These transporters actively pump out chemotherapeutic drugs, reducing their intracellular concentration and leading to decreased treatment efficacy [35]. In this context, exosomal lncRNAs have been shown to influence the expression of drug efflux pumps through various mechanisms, thereby modulating drug resistance [36]. For instance, lncRNAs within exosomes, such as linc-ROR and linc-VLDLR, enhance the expression of the pivotal efflux protein ABCG2, thereby promoting hepatocellular carcinoma’s resistance to chemotherapeutics like sorafenib and doxorubicin [37]. Exosomal lncRNAs can also act as competitive endogenous RNAs (ceRNAs), sequestering and blocking the function of miRNAs to prevent the degradation of target mRNAs, thereby indirectly regulating drug resistance pathways. For example, lncRNA H19 promotes colorectal cancer resistance to oxaliplatin by sequestering miR-141 [38]. Exosomal lncRNAs can also enhance the glycolytic capacity of recipient cells, thereby increasing the energy available for drug efflux pumps and reducing drug sensitivity [39]. These mechanisms emphasize the complex interactions between exosomal lncRNAs and the processes controlling drug efflux and cellular sensitivity.

Beyond their roles in cell communication, angiogenesis, and drug efficacy, exosomal lncRNAs can also reshape tumor growth states by modifying the tumor microenvironment, promoting epithelial-mesenchymal transition (EMT), and facilitating tumor proliferation and migration (Table 1), thereby indirectly influencing drug resistance. This offers a novel perspective on resistance mechanisms.

**Table 1:** The association between long non-coding RNAs(lncRNAs) and drug resistance in various types of cancer

Cancer type	Expression	Lncrna	Numbers of clinical samples	Clinical features			Ref
				Diagnosis	Prognosis	Clinical significance	
Colorectal cancer (CRC)	Up	CACClnc	53 colorectal cancer tissues from patients who underwent surgery	CACClnc expression was positively correlated with recurrence and AJCC stage in CRC	Recurrence rate in the high-risk group (70%) was significantly higher than the low-risk group (16%)	Pre-therapy plasma exosomal cacclnc may predict chemotherapy responses and guide treatment strategies for CRC patients	[43]
	Up	FAL1	CRC issues and adjacent normal specimens were sampled from 98 patients	Lnc-FAL1 level was overexpressed in CRC tissues	Higher lnc-FAL1 expression level was associated with a poorer overall survival and disease-free survival inCRC patients	Lnc-FAL1 expression is linked to tumor size, regional lymph node metastasis, distant metastasis, and recurrence	[44]
	Up	CCAL	15 pairs of CRC tissue samples and adjacent non-cancerous tissues from patients	–	–	CCAL is significantly overexpressed in CRC tissues compared to paired adjacent tissues	[12]
	Up	H19	Isolated primary normal fibroblasts (NFs) and CAFs from 10 paired tumor and adjacent normal tissues from CR patients	H19 expression significantly increases in early stages of CRC (stage i, ii, and iii) and is higher in metastatic stage iv compared to non-metastatic stage iv cases	–	H19 is indicative of cancer grading	[15]
	Up	SNHG11	27 pairs of cancer and adjacent normal tissues from bevacizumab treated CRC patients	SNHG11 was upregulated in colon adenocarcinoma compared to normal controls, especially in bevacizumab sensitive CRC tissues	SNHG11 knockdown inhibited xenograft tumor volume and tumor weight compared to related controls	SNHG11 might provide a novel insight for hindering bevacizumab resistance in CRC	[14]
	Up	PGM5-AS1	62 pairs of cancer and adjacent normal tissues from CRC patients	PGM5‐AS1 is lowly expressed in oxaliplatin‐resistant CRC tissues	PGM5‐AS1 inhibits proliferation, metastasis, and acquired oxaliplatin resistance of colon cancer cells in vivo	Engineered exosomes co‐delivering PGM5‐AS1 and oxaliplatin reverses drug resistance in CRC	[45]
Esophageal cancer (EC)	Up	MIAT	48 patients (30 males and 18 female) who received PTX treatment following resection of ESCC	MIAT is significantly upregulated in serum-derived exosomes from PTX non-responders	–	Silencing MIAT delayed EC cell growth and reduced their resistance to PTX in vivo	[46]
	Up	POU3F3	Included 138 patients with locally advanced ESCC undergo ccrt and 78 ESCC patients with postoperative recurrence	In ESCC patients, plasma exosomal lncrna POU3F3 levels were significantly elevated	Pou3f3 levels decreased and were later upregulated in tumor progression, suggesting its potential as a prognostic biomarker	In ESCC patients, plasma exosomal lncrna POU3F3 levels were significantly elevated	[47]
Gastric cancer (GC)	Up	MALAT1	–	MALAT1 serves as a biomarker for early cancer diagnosis through blood or other fluid samples	Its high expression correlates with poor prognosis, predicting survival and recurrence risk	MALAT1 sirna effectively suppresses malat1 expression in tumor cells and tams, enhancing chemotherapy efficacy in mouse tumor models	[48]
	Down	DACT3-AS1	Acquired 93 GC tissues from postoperative samples at shandong cancer hospital between may 2019 and october 2020	–	–	Exosomal DACT3-AS1 may serve as a potential biomarker for diagnosis and therapeutic targeting in GC	[49]
	Up	HOTTIP	A total of 58 serum samples were enrolled	Exosomal HOTTIP abundance is increased in cisplatin-resistant patients	Higher proportion of non-responders to chemotherapy in the high HOTTIP level group	Serum exosomal HOTTIP may serve as a promising diagnostic biomarker for GC patients	[50]
	Up	CRNDE	35 pairs of cancerous tissues and adjacent normal tissues from GC patients	35 pairs of cancerous tissues and adjacent normal tissues	LncRNA CRNDE induces CDDP resistance in GC cells	Silencing of LncRNA CRNDE can restore the sensitivity of GC cells to CDDP, which is conducive to the treatment of GC	[51]
Pancreatic cancer (PC)	Up	UCA1	35 pancreatic cancer patients’ pancreatic cancer tissues and adjacent normal tissues	High expression of exosomal lncRNA UCA1 promoted the malignant phenotypes, and augmented the Gem resistance of pancreatic cancer	Knockdown of lncRNA UCA1 inhibited tumorigenesis in mice xenografting with pancreatic cancer cells	Targeting lncRNA UCA1 can treat exosom-mediated angiogenesis of hypoxic pancreatic cancer cell origin	[52]
	Up	ROR	–	The levels of LncROR were higher in the chemoresistant PANC1 cells	LncROR promotes dryness and chemotherapy resistance of PC cells	This study suggested that exosomal LncROR may serve as a candidate target of chemotherapy for PC	[53]
Hepatocellular cancer (HCC)	Up	ROR	Hepg2 and Plc-prf5 cells were obtained from American type	Lncrna ROR is increased by over twofold in malignant cells compared to non-malignant cells	–	Targeting these intercellular signaling mechanismsmay improve responses to conventional therapies for HC	[54]
	Up	H19	12 mice were divided into two groups and injected with propofol and Vector or Over H19-treated Huh7 cell-derived exosomes	Exosome H19 from Huh7 cells enhances the malignant potential of HCC cells	Exosome H19 promotes the growth of HCC tumors treated with propofol	–	[55]

### Role of lncRNAs in resistance to targeted therapies in gastrointestinal cancers

In addition to their established role in modulating chemotherapy resistance, lncRNAs have also emerged as crucial regulators of resistance to molecularly targeted therapies in gastrointestinal (GI) cancers. These therapies, including agents targeting EGFR, VEGFR, and HER2, are widely employed in colorectal, gastric, and hepatocellular carcinomas. Increasing evidence indicates that specific lncRNAs can bypass or attenuate the effects of such targeted treatments. For instance, lncRNA UCA1 has been reported to induce resistance to EGFR inhibitors in colorectal cancer by activating the AKT/mTOR signaling pathway [40]. Similarly, HOTAIR contributes to trastuzumab resistance in HER2-positive gastric cancer via epigenetic suppression of tumor suppressor genes and promotion of epithelial-mesenchymal transition [41]. Moreover, lncRNAs such as H19 and MALAT1 have been implicated in resistance to anti-VEGF therapies, facilitating angiogenesis and tumor microenvironment remodeling [42]. These lncRNAs support oncogenic signaling and promote cell survival despite therapeutic inhibition, highlighting their critical role in acquired resistance mechanisms. A comprehensive understanding of these pathways is essential for overcoming resistance and optimizing targeted therapy efficacy in GI malignancies.

### Colorectal cancer

Colorectal cancer is the third most commonly diagnosed cancer globally and ranks as the fourth leading cause of cancer-related mortality [56]. Despite advancements in screening, surgical interventions, radiation therapy, and chemotherapy, over 40% of colorectal cancer patients still succumb to the disease, primarily due to metastasis and recurrence, as residual cancer cells cannot be fully eradicated or controlled. One of the primary factors contributing to this high mortality rate is the development of chemoresistance to multiple therapeutic agents [57].

Oxaliplatin, a platinum-based chemotherapeutic agent, is widely used for treating solid tumors, particularly colorectal cancer. It exerts its cytotoxic effects by forming platinum–DNA adducts in rapidly dividing cells. These adducts induce DNA crosslinking, inhibiting replication and transcription, ultimately leading to cell death [58]. However, the development of resistance to oxaliplatin poses a significant clinical challenge, often necessitating combination therapies and reducing patient survival rates [59]. Exosomal lncRNAs have been implicated in the development of oxaliplatin resistance in colorectal cancer.

### CACClnc

Recent studies have identified a novel lncRNAs, (CACClnc), which has been shown to confer oxaliplatin resistance in colorectal cancer cells. CACClnc expression is upregulated in oxaliplatin-resistant colorectal cancer and in patients experiencing cancer recurrence, highlighting its association with chemoresistance and disease progression.

The mechanisms underlying CACClnc-mediated chemoresistance have been elucidated in subsequent studies. CACClnc interacts with the splicing factor Y-box binding protein 1 (YB1) and U2 auxiliary factor 65 (U2AF65), a subunit of the U2AF splicing factor. This interaction regulates the alternative splicing of RAD51 mRNA. RAD51 is a critical protein involved in homologous recombination repair, and its aberrant alternative splicing is one of the mechanisms through which oxaliplatin exerts cytotoxicity. However, CACClnc-mediated regulation of RAD51 splicing disrupts this process, facilitating homologous recombination and promoting oxaliplatin resistance in cancer cells.

Furthermore, CACClnc expression is regulated through epigenetic modifications. Decreased N6-methyladenosine (m6A) modification in CACClnc RNA has been implicated in its upregulation within cancer cells. Similar to other lncRNAs involved in chemoresistance, CACClnc can be incorporated into exosomes and transferred to neighboring cells, thereby propagating oxaliplatin resistance throughout the tumor microenvironment [43].

### CCAL, H19 and FAL1

Several lncRNAs originating from cancer-associated fibroblasts (CAFs), such as CCAL and H19, have also been found to play key roles in the development of oxaliplatin resistance in colorectal cancer. And the clinical evidence shows that high serum CCAL and H19 in patients are associated with poor prognosis and chemoresistance to oxaliplatin.

Notably, H19, previously discussed in the context of cancer development, has been shown to contribute to colorectal cancer chemoresistance through distinct mechanisms. In colorectal cancer, H19 promotes cancer cell stemness and resistance to oxaliplatin by acting as a ceRNA that mute miR-141, a microRNA known to suppress cancer stemness by targeting the β-catenin to block the WNT/β-catenin pathway [60]. Additionally, stromal CAF-derived H19 can be transferred to cancer cells, further contributing to the spread and progression of chemoresistance within the tumor [15].

Similarly, CCAL, another lncRNA produced by CAFs, is upregulated in oxaliplatin-resistant colorectal cancer. CCAL promotes chemoresistance by activating the mRNA-stabilizing protein human antigen R (HuR) [12]. HuR binds and stabilizes the β-catenin mRNAs [61]. The activation of HuR subsequently increases β-catenin levels, suppressing apoptosis and diminishing oxaliplatin efficacy. As with H19, CCAL can also be transferred from CAFs to cancer cells via exosomes, facilitating the propagation of chemoresistance throughout the tumor [12].

Another long non-coding RNA, FAL1, has been found to be upregulated in colorectal cancer. The dysregulation of FAL1 has been implicated in the tumorigenesis and progression of various malignancies through multiple mechanisms [62, 63]. A recent study involving 98 colorectal cancer samples demonstrated that CAF-secreted, exosome-transferred FAL1 contributes to chemoresistance to oxaliplatin. This occurs through FAL1 acting as a scaffold that facilitates the interaction between Beclin1 and TRIM3, promoting TRIM3-mediated polyubiquitination and degradation of Beclin1. Consequently, this process suppresses oxaliplatin-induced autophagic cell death in colorectal cancer cells [44].

### SNHG11

Bevacizumab is a widely used therapeutic agent for the treatment of colorectal cancer, particularly in cases of metastasis. It is a monoclonal antibody that targets vascular endothelial growth factor (VEGF), thereby inhibiting tumor growth and EMT [64].

Small nucleolar RNA host gene 11 (SNHG11) is a lncRNA that has been found to be upregulated in bevacizumab-resistant colorectal cancer. Studies have demonstrated that elevated SNHG11 expression is associated with increased levels of cyclin D1 and multidrug resistance protein 1 (MDR1) [14]. SNHG11 exerts its effects by downregulating miR-1207-5p, a microRNA shown to inhibit bevacizumab resistance in colorectal cancer cells. Mir-1207-5p normally targets ATP-binding cassette subfamily C member 1 (ABCC1), a well-established mediator of multidrug resistance by introducing efflux pumps to cancer cells, to reduce its effect in chemoresistance development [65]. Similar to other lncRNAs mentioned earlier, SNHG11 has been shown to be transferred between cells via exosomes, contributing to the spread of chemoresistance and cancer advancement [14].

### Gastric cancer

Gastric cancer remains a major global health burden, ranking as the second leading cause of cancer-related mortality worldwide. The overall 5-year survival rate for gastric cancer patients is less than 25%, primarily due to the fact that most cases are diagnosed at advanced stages when prognosis is poor [66]. Advanced gastric cancer is typically associated with a high degree of metastasis and treatment challenges. As a result, chemotherapy is the standard treatment modality for these patients. However, the development of chemoresistance remains a significant clinical hurdle, rendering chemotherapy less effective or, in some cases, completely ineffective [67]. Increasing evidence suggests that lncRNAs play a crucial role in mediating chemoresistance in gastric cancer to multiple therapeutic agents [68].

### DACT3-AS1

Oxaliplatin is commonly employed in the treatment of gastric cancer. One notable lncRNA associated with oxaliplatin resistance in gastric cancer is disheveled binding antagonist of beta catenin 3 antisense 1 (DACT3-AS1). In oxaliplatin-resistant gastric cancer cells, a significant downregulation of DACT3-AS1 has been observed. DACT3-AS1 is secreted by cancer-associated fibroblasts and is incorporated into exosomes to be transferred to cancer cells. In cancer cells, it exhibits tumor-suppressive effects. The primary mechanism by which DACT3-AS1 exerts its effects is through the miR-181a-5p/SIRT1 axis. Normally, miR-181a-5p promotes tumorigenesis by inhibiting SIRT1, a gene involved in tumor suppression and cellular homeostasis. However, DACT3-AS1 negatively regulates miR-181a-5p, thereby alleviating its suppression of SIRT1. This action reduces cancer cell proliferation, invasion, and metastasis. Furthermore, DACT3-AS1 plays a pivotal role in restoring oxaliplatin sensitivity by inducing ferroptosis, a form of regulated cell death driven by iron-dependent lipid peroxidation. Thus, the downregulation of DACT3-AS1 in gastric cancer not only promotes tumor progression but also facilitates the development of resistance to oxaliplatin, highlighting its dual role in both tumor biology and chemoresistance. Clinically, high levels of DACT3-AS1 in serum exosomes were associated with poor prognosis of gastric cancer and insensitivity of patients to oxaliplatin [49].

### HOTTIP, FGD5-AS1 and CRNDE

Cisplatin, another platinum-based chemotherapeutic agent, is widely used in the treatment of gastric cancer. Like oxaliplatin, cisplatin functions by forming DNA adducts, thereby disrupting DNA replication and transcription, leading to cell death. However, as with oxaliplatin, the development of cisplatin resistance in gastric cancer significantly reduces treatment efficacy [69].

Recent studies have identified several lncRNAs, HOXA transcript at the distal tip (HOTTIP), FGD5-AS1 and CRNDE, as being upregulated in cisplatin-resistant gastric cancer cells. Upregulation of HOTTIP and FGD5-AS1 in cisplatin-resistant cells appears to be driven by a combination of epigenetic and transcriptional stimuli. Hypoxia, a hallmark of advanced tumors, stabilizes HIF-1α, which directly binds hypoxia-response elements (HREs) in the HOTTIP promoter, increasing its transcription [50]. Likewise, NF-κB activation downstream of chronic DNA damage fosters FGD5-AS1 expression by recruiting p300/CBP to its enhancer region [70].

HOTTIP acts as a ceRNA by sponging miR-218 while FGD5-AS1 sponges miR-195, two microRNAs known for its tumor-suppressive function [50, 71]. MiR-218 typically inhibits the expression of high-mobility group AT-hook 1 (HMGA1), a protein that plays a critical role in chromatin remodeling and transcriptional regulation to facilitate cell proliferation [72]. And miR-195 can target various signaling molecules including cyclin D1, cyclin E1, survivin, BCL2 and VEGF to suppress a series of cancerogenic processes. By sequestering miR-218 and miR-195, HOTTIP and FGD5-AS1 promote tumor cell proliferation, migration, and invasion. This mechanism facilitates not only the progression of gastric cancer but also the acquisition of cisplatin resistance [73, 74].

Both HOTTIP and FGD5-AS1 are enriched in extracellular vesicles owing to specific sequence motifs, EXOmotifs, that bind the RNA-binding protein hnRNPA2B1, facilitating their loading into exosomes [28, 75]. Exosomal transfer of these lncRNAs to naïve gastric cancer cells induces a resistant phenotype, as recipient cells acquire miR-218 and miR-195 depletion, HMGA1 and PI3K/AKT hyperactivation, and increased DNA repair capacity [50, 70]. This intercellular dissemination of resistance underscores a paracrine mechanism by which small populations of resistant cells educate the tumour microenvironment.

Moreover, the exosome-mediated transfer of HOTTIP facilitates heterogeneity of tumor cells, further complicating treatment strategies and contributing to poor clinical outcomes [50, 70].

Another lncRNA, CRNDE, is also found to be upregulated in cisplatin-resistant gastric cancer. Unlike HOTTIP and FGD5-AS1, CRNDE is produced and secreted by TAMs. TAMs secrete IL-6 and TGF-β, which engage STAT3 and SMAD3 to induce CRNDE transcription in both macrophages and adjacent carcinoma cells [51].

CRNDE acts by facilitating the effect of neural precursor cell expressed developmentally downregulated protein 4–1 (NEDD4-1), an E3 ubiquitin ligase for mediated phosphatase and tensin homolog (PTEN). PTEN is a well-demonstrated tumor suppressor which counteracts PI3K-AKT pathway [76]. The upregulated CRNDE will contribute to NEDD4-1-mediated PTEN ubiquitination, preventing its inhibitory effect on cell proliferation and survival of gastric cancer cells. Moreover, study has also shown that TAM-derived CRNDE are transferred to cancer cells in exosome to spread the development of cisplatin-resistance in tumor [51].

### Esophageal cancer

Esophageal cancer is the eighth most common type of cancer worldwide with a mortality that ranks sixth place among all cancers. According to the different origins of cancer cells, estrogen cancer can be roughly divided into two subtypes, squamous cell carcinoma and adenocarcinoma [77]. The symptoms of esophageal cancer often do not appear until the disease is advanced and examinations for esophageal cancer are still in development, thus, patients with esophageal cancer are usually being diagnosed at late stage [78]. Thus, chemotherapy is widely used combining with other procedures in the treatment of esophageal cancer. In this case, the development of chemoresistance has become a significant barrier for esophageal cancer treatment.

### MIAT

Paclitaxel is a common chemotherapeutic agent for the treatment of esophageal cancer in combination with other chemotherapies. It belongs to taxanes and works by interfering with the normal function of microtubules to block the division of cells. The use of paclitaxel can largely relieve the symptoms and promote the effect of other drugs [79]. An exosome-encapsulated lncRNA myocardial infarction-associated transcript (MIAT), is found to be produced by esophageal cancer cells and indicated to mediate the development of chemoresistance to paclitaxel. The level of MIAT is found to be upregulated in paclitaxel-resistant cancer cells both in clinical serum and cell lines underwent paclitaxel administration. The mechanism for MIAT to exert chemoresistance to cancer cells is that MIAT can recruit a transcription factor, TATA-box binding protein-associated Factor 1 (TAF1), in the promoter region of sterol regulatory element binding transcription factor 1 (SREBF1) and enhance the expression of SREBF1 [46]. SREBF1 has previously been indicated as a hallmark of cancer, and its overexpression has been associated with several mechanisms for tumorigenesis, including the promotion of lipid synthesis, facilitation of tumor survival under stress and dysregulation of cell metabolism [80]. Thus, MIAT-induced SREBF1 overexpression can largely facilitate tumor survival and development of chemoresistance. Additionally, exosomal MIAT is also shown to be secreted from the cancer cells and transferred into other cancer cells to spread the chemoresistance to paclitaxel [46].

### POU3F3

Another lncRNA secreted by esophageal tumor cells, POU3F3, is also found to be upregulated in advanced esophageal cancer with resistance to cisplatin, another chemotherapeutic drug widely used for esophageal cancer treatment. But the effect of POU3F3 on chemoresistance development is not directly exerted into the cancer cells. Instead, POU3F3 promotes tumor advancement and chemoresistance by transforming normal fibroblasts into cancer-associated fibroblasts. POU3F3 from cancer cells can be transferred to normal fibroblasts in the exosomes, subsequently transform and activate fibroblasts into cancer-associated fibroblasts. The activated fibroblasts can not only promote the invasion and migration of esophageal cancer cells, but also can secrete interleukin 6 [47]. It has been previously demonstrated that the interference of cytokine secretion from cancer-associated fibroblasts is a key mechanism for the development of chemoresistance in cancer [81]. And studies have shown that the secretion of interleukin 6 can facilitate the resistance of cisplatin in esophageal cancer [82].

### Hepatocellular cancer

Hepatocellular carcinoma is the most common form of primary liver cancer, frequently arising from chronic liver conditions such as hepatitis B and C infections, liver cirrhosis, and non-alcoholic fatty liver disease [83]. Similar to other gastrointestinal tract cancers, hepatocellular carcinoma is often asymptomatic in its early stages, making early detection difficult. As a result, chemotherapy is commonly employed in advanced stages, often in conjunction with surgical resection, ablation, or other therapeutic strategies to improve prognosis and prevent recurrence [84]. However, hepatocellular carcinoma is known to exhibit a high level of resistance to many chemotherapeutic agents, presenting a significant challenge in treatment [85].

### H19

Exosomal H19 has been shown to exacerbate hepatocellular carcinoma progression in response to propofol treatment. While propofol is widely utilized as a fast-acting intravenous anesthetic, emerging studies indicate that it also exhibits potential anti-tumor properties [86]. However, further research reveals that in hepatic cancer cells, upregulated H19 functions as a ceRNA by sponging miR-520a-3p, thereby relieving its suppressive effect on LIM domain kinase 1 (LIMK1), an enzyme that promotes HCC cell proliferation, metastasis, and inhibits apoptosis [55]. Consequently, elevated H19 levels facilitate cancer progression through the disinhibition of LIMK1, contributing to tumor advancement.

### LncROR

Transforming Growth Factor-β (TGF-β) orchestrates chemoresistance in hepatocellular carcinoma (HCC) by upregulating the long intergenic non-coding RNA LncROR and facilitating its extracellular dissemination. In sorafenib- and doxorubicin-resistant HCC cell lines generated via stepwise drug exposure, CCK-8, EdU and qRT-PCR analyses demonstrated significantly higher LncROR levels in resistant (e.g., SNU449) versus sensitive (e.g., Huh7) cells, correlating with increased IC₅₀ values for both agents [87–89]. Exogenous TGF-β treatment was shown to decrease sensitivity to both sorafenib and doxorubicin, while enhancing EV release and selectively enriching LncROR within these vesicles [54, 90]. Mechanistically, LncROR promotes epithelial-mesenchymal transition (EMT) by upregulating TWIST1 and downregulating E-cadherin, with concomitant vimentin induction, thus enhancing invasion and further resistance [54, 88, 91]. Additionally, TGF-β–mediated enrichment of LncROR in exosomes increases CD133⁺ cancer stem cell populations and inhibits p53-dependent cell death, propagating a stem-like, chemoresistant phenotype throughout the tumor microenvironment [90, 92].

Moreover it has been shown that both H19 and LncROR can be transferred between cells via exosomes, leading to the increased activation of LIMK1 and TGFβ, respectively, in neighboring cells and thereby contributing to the spread of chemoresistance across the tumor microenvironment [54, 55].

### Pancreatic cancer

Pancreatic cancer ranks among the most lethal and aggressive malignancies, characterized by a high mortality rate. One of the key challenges in managing pancreatic cancer is that it is often diagnosed at advanced stages, where curative surgical interventions are rarely feasible due to the tumor's proximity to vital organs such as the intestines and gallbladder. This anatomical complexity, coupled with the tumor's aggressive nature, renders surgical options impractical in most cases [93]. As a result, chemotherapy and palliative treatments are the primary therapeutic strategies for managing pancreatic cancer.

### LncROR

Hypoxia, a condition of reduced oxygen availability, has been widely implicated in the progression and aggressiveness of various cancers [94]. Recent findings also suggest that hypoxia plays a pivotal role in promoting chemoresistance in pancreatic cancer. Specifically, one study demonstrated that hypoxic conditions increased the resistance of pancreatic cancer cells to gemcitabine, a first-line chemotherapeutic agent that inhibits DNA synthesis [95]. Under 1% O₂, pancreatic cancer cells release increased numbers of exosomes selectively enriched in LncROR, as confirmed by nanoparticle tracking and vesicle RNA profiling [53].

The study further revealed that hypoxia induces the production and secretion of exosomes containing the LncROR from pancreatic cancer cells. LncROR has been shown to inhibit the Hippo/Yes-associated protein (Hippo/YAP) signaling pathway, a key regulator of cell proliferation and apoptosis [53]. Upon uptake by recipient cells, exosomal LncROR binds components of the Hippo pathway, reducing MST1/2 and LATS1 phosphorylation, thereby preventing YAP Ser127 phosphorylation and allowing YAP nuclear translocation and activation of TEAD-dependent targets such as CTGF and CYR61, which drive proliferation and survival [96]. Simultaneously, LncROR acts as a competing endogenous RNA for miR-145, derepressing Nanog and OCT4 expression to expand the CD133⁺ cancer stem cell pool and inhibit gemcitabine-induced apoptosis and G₁/S arrest, resulting in a 30–50% increase in gemcitabine IC₅₀ values in functional assays [97, 98]. This dual mechanism underscores the pivotal role of hypoxia-elicited, exosome-mediated LncROR transfer in promoting chemoresistance and tumor progression in pancreatic cancer [53].

### Overview of LncROR in digestive cancers

**Fig. 2:**
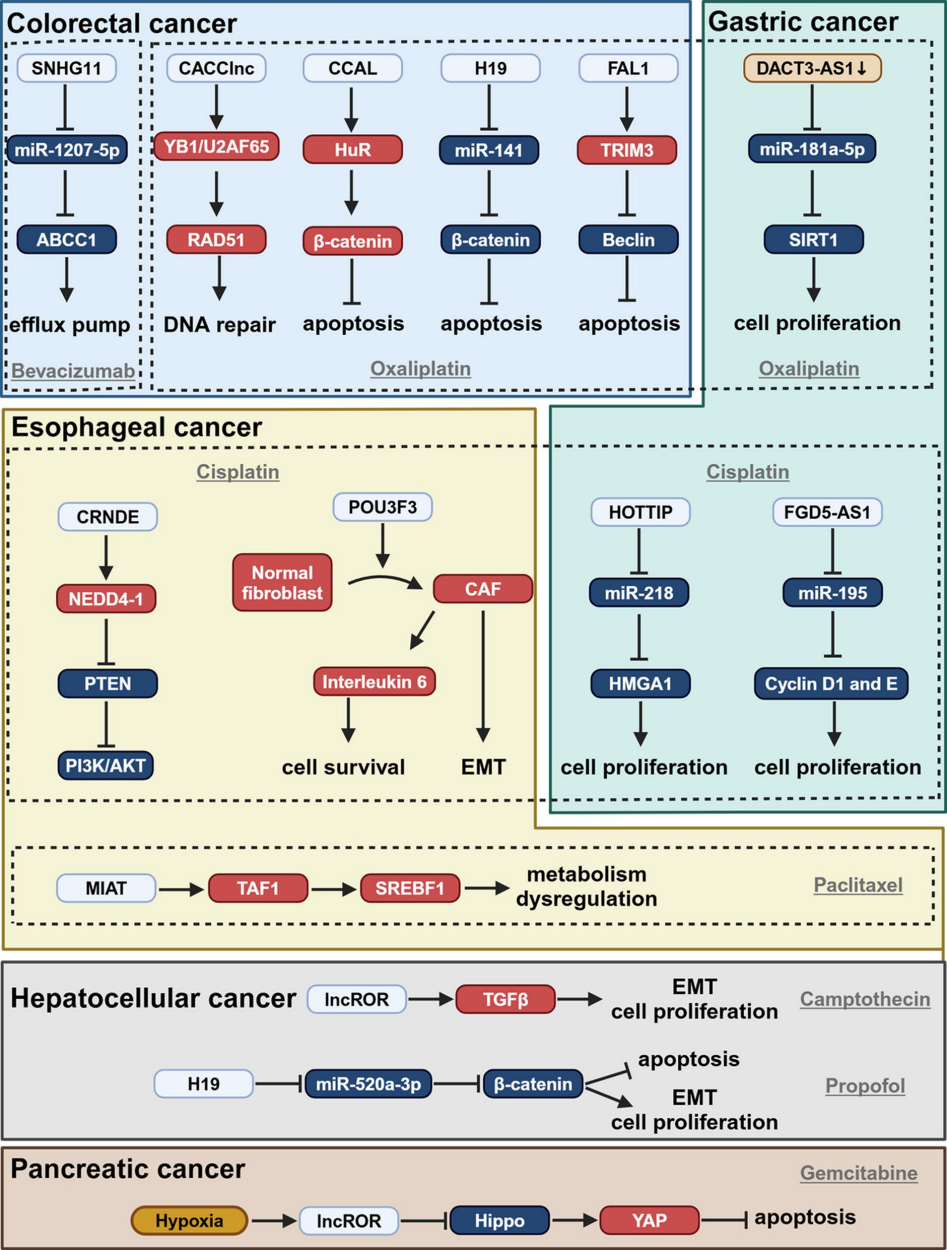
Mechanistic roles of lncRNAs in GI cancers. This figure illustrates the involvement of various long non-coding RNAs (lncRNAs) in the regulation of critical signaling pathways across different GI cancers, including colorectal, gastric, esophageal, hepatocellular, and pancreatic cancers. In colorectal cancer, lncRNAs such as SNHG11, CACClnc, CCAL, H19, and FAL1 modulate pathways related to efflux pump regulation (ABCC1), DNA repair (RAD51), apoptosis (β-catenin, Beclin), and drug resistance (Bevacizumab). Similarly, in gastric cancer, lncRNAs DACT3-AS1 and FGD5-AS1 promote cancer cell proliferation via SIRT1 and Cyclin D1/E pathways, with implications for resistance to drugs like Oxaliplatin and Cisplatin. Esophageal cancer features lncRNAs such as MIAT and POU3F3, which influence metabolic dysregulation, EMT (epithelial-mesenchymal transition), and cell survival through interactions with key proteins like TAF1, SREBF1, and Interleukin-6. Hepatocellular and pancreatic cancers demonstrate the regulation of EMT and apoptosis by lncRNAs LncROR and associated pathways (TGFβ, Hippo/YAP), which also influence response to chemotherapeutic agents like Camptothecin and Gemcitabine. Created in BioRender

LncROR has been implicated as a central driver of chemoresistance across digestive tract malignancies by coordinating drug efflux, EMT, and cancer stem cell (CSC). In gastric cancer, LncROR is significantly upregulated in adriamycin- and vincristine-resistant cell lines, where it enhances multidrug resistance–associated protein 1 (MRP1) expression and activates TWIST1- and β-catenin–mediated EMT, correlating with poorer patient survival [99]. In HCC, hypoxia or TGF-β stimulation enriches LncROR within tumor-derived exosomes; uptake of these vesicles by naïve HCC cells elevates LncROR levels, attenuates sorafenib- or doxorubicin-induced apoptosis, and expands the CD133⁺ CSC pool via TWIST1 activation and p53 pathway inhibition [54]. Similarly, in pancreatic ductal adenocarcinoma, hypoxia-elicited exosomes selectively load LncROR, which in recipient cells suppresses the Hippo kinase cascade (decreasing MST1/2 and LATS1 phosphorylation), promotes YAP nuclear translocation and TEAD-dependent transcription of CTGF and CYR61, and concurrently sponges miR-145 to derepress Nanog and OCT4—collectively enhancing gemcitabine resistance, inhibiting apoptosis, and reinforcing CSC characteristics [100]. Across these cancers, LncROR unifies EMT induction with competing endogenous RNA mechanisms and exosomal transfer to sustain drug efflux transporter expression and CSC phenotypes, highlighting the TGF-β/LncROR/exosome axis as a promising therapeutic target to overcome chemoresistance in gastric, hepatic, and pancreatic malignancies (Fig. 2**)**.

## Mechanisms of chemoresistance mediated by exosomal lncRNAs

Chemoresistance, or the ability of cancer cells to resist the effects of chemotherapeutic agents, is a significant obstacle in cancer treatment. One emerging area of research has demonstrated that exosomal lncRNAs play a pivotal role in mediating chemoresistance across various cancer types. These lncRNAs are secreted within exosomes—small vesicles that facilitate intercellular communication—and can be transferred between cells, contributing to resistance mechanisms in surrounding cancer cells or within the tumor microenvironment. In this section, we will examine the different mechanisms through which exosomal lncRNAs mediate chemoresistance, highlighting the critical molecular pathways involved.

### DNA damage repair and homologous recombination

One of the primary mechanisms through which chemotherapeutic agents induce cancer cell death is by inducing DNA damage, often overwhelming the cell repair capabilities. However, cancer cells can develop resistance by enhancing their DNA repair mechanisms, a process in which exosomal lncRNAs have been found to play an important role.

For instance, in colorectal cancer, the lncRNA CACClnc promotes oxaliplatin resistance by facilitating DNA repair. It interacts with splicing factors such as YB1 and U2AF65 to regulate the alternative splicing of RAD51, a key protein in homologous recombination repair [43]. This modulation of RAD51 splicing allows cancer cells to better repair the DNA damage caused by oxaliplatin, thus promoting resistance. Additionally, CACClnc can be transferred via exosomes to neighboring cells, spreading the chemoresistance throughout the tumor microenvironment.

In glioblastoma, the lncRNA SBF2-AS1 acts as a ceRNA for miR-151a-3p, preventing the latter from inhibiting XRCC4, a crucial protein involved in DNA double-strand break repair. The overexpression of SBF2-AS1 enhances DNA repair mechanisms, thereby reducing the effectiveness of temozolomide, an alkylating agent used in glioblastoma treatment. This chemoresistance can be propagated to surrounding cells through exosomal transfer of SBF2-AS1, spreading the resistance within the tumor [101].

### Epigenetic modifications and chromatin remodeling

Another mechanism through which cancer cells develop chemoresistance involves epigenetic changes, which alter gene expression without changing the underlying DNA sequence. Exosomal lncRNAs can influence these processes by interacting with chromatin remodelers or by sponging miRNAs that regulate epigenetic factors.

In gastric cancer, the lncRNA HOTTIP is upregulated in cisplatin-resistant cells. HOTTIP functions as a ceRNA by sponging miR-218, a tumor-suppressive miRNA that normally inhibits the expression of HMGA1, a protein involved in chromatin remodeling. By sequestering miR-218, HOTTIP upregulates HMGA1, enhancing cancer cell proliferation and invasion while promoting cisplatin resistance. Exosomal HOTTIP can be transferred to neighboring cells, further spreading chemoresistance within the tumor microenvironment [50].

Similarly, in glioblastoma, the lncRNA HOTAIR is upregulated in temozolomide-resistant cells. HOTAIR sponges miR-519a-3p, which normally inhibits ribonucleotide reductase M1 (RRM1), an enzyme essential for DNA synthesis and repair. By preventing the inhibition of RRM1, HOTAIR promotes DNA replication and repair, leading to temozolomide resistance. This exosome-mediated transfer of HOTAIR contributes to the spread of chemoresistance across glioblastoma cells [102].

### Tumor microenvironment and immune modulation

Exosomal lncRNAs also play a critical role in modulating the tumor microenvironment, which includes not only cancer cells but also surrounding stromal cells, immune cells, and fibroblasts. By altering the behavior of these non-cancerous cells, exosomal lncRNAs contribute to the development and spread of chemoresistance.

In esophageal cancer, the lncRNA POU3F3 is secreted by tumor cells and incorporated into exosomes, which are then taken up by fibroblasts. POU3F3 promotes the transformation of normal fibroblasts into CAFs, which are known to secrete cytokines that support cancer cell survival and chemoresistance. Specifically, CAFs activated by exosomal POU3F3 secrete interleukin-6, a cytokine that promotes resistance to cisplatin, a common chemotherapeutic drug for esophageal cancer [47].

In colorectal cancer, exosomal lncRNAs like CCAL and H19, which are produced by CAFs, also play a significant role in modulating the tumor microenvironment. CCAL promotes chemoresistance by stabilizing β-catenin mRNA via the RNA-binding protein HuR. This stabilization enhances β-catenin signaling, which suppresses apoptosis and contributes to resistance to oxaliplatin. Similarly, H19 acts as a ceRNA for miR-141, a microRNA that suppresses cancer stemness by targeting the WNT/β-catenin pathway. By inhibiting miR-141, H19 promotes cancer stemness and resistance to oxaliplatin [12, 15].

Another lncRNA, temozolomide associated lncRNA in GBM recurrence (TALC), is also found to be upregulated in temozolomide-resistant glioblastoma. TALC is produced by tumor-associated macrophages (TAM), specifically microglia. And studies have demonstrated that TALC from microglia can be incorporated into exosomes and secreted to the tumor cells. TALC mediates chemoresistance to cancer cells by elevating C5 expression. C5 is a DNA damage repair-related cytokine and an upregulated C5 can facilitate the DNA repair process induce by temozolomide, leading to chemoresistance. Further, the results have also shown that TALC can also promote the M2 polarization of microglia, which is anti-inflammatory and can prevent the apoptosis of temozolomide-induced cell death [103].

### Reduced apoptosis and ferroptosis

Ferroptosis, a form of regulated cell death characterized by iron-dependent lipid peroxidation, is an emerging target for cancer therapies [49]. Exosomal lncRNAs have been found to influence ferroptosis and metabolic pathways in cancer cells, thereby contributing to chemoresistance [46].

In gastric cancer, the lncRNA DACT3-AS1 is downregulated in oxaliplatin-resistant cells. DACT3-AS1 is secreted by CAFs and incorporated into exosomes, where it is transferred to cancer cells. It exerts tumor-suppressive effects by negatively regulating miR-181a-5p, which in turn relieves the inhibition of SIRT1, a gene involved in ferroptosis. By restoring ferroptosis, DACT3-AS1 sensitizes cancer cells to oxaliplatin. However, the downregulation of DACT3-AS1 in resistant cells reduces ferroptosis, facilitating tumor survival and chemoresistance [49].

### Alternative signaling pathways

Metabolic reprogramming is another important mechanism through which cancer cells evade the cytotoxic effects of chemotherapy [104]. Exosomal lncRNAs can dysregulate the intracellular metabolism by introducing alternative signaling pathways inside the cells [105].

In esophageal cancer, the exosomal lncRNA MIAT promotes paclitaxel resistance by recruiting the transcription factor TAF1 to the promoter of SREBF1, a gene involved in lipid synthesis and cell metabolism [106]. The upregulation of SREBF1 promotes cancer cell survival under stress and contributes to chemoresistance. MIAT is secreted within exosomes and transferred to other cancer cells, spreading resistance to paclitaxel within the tumor [46].

**Fig. 3:**
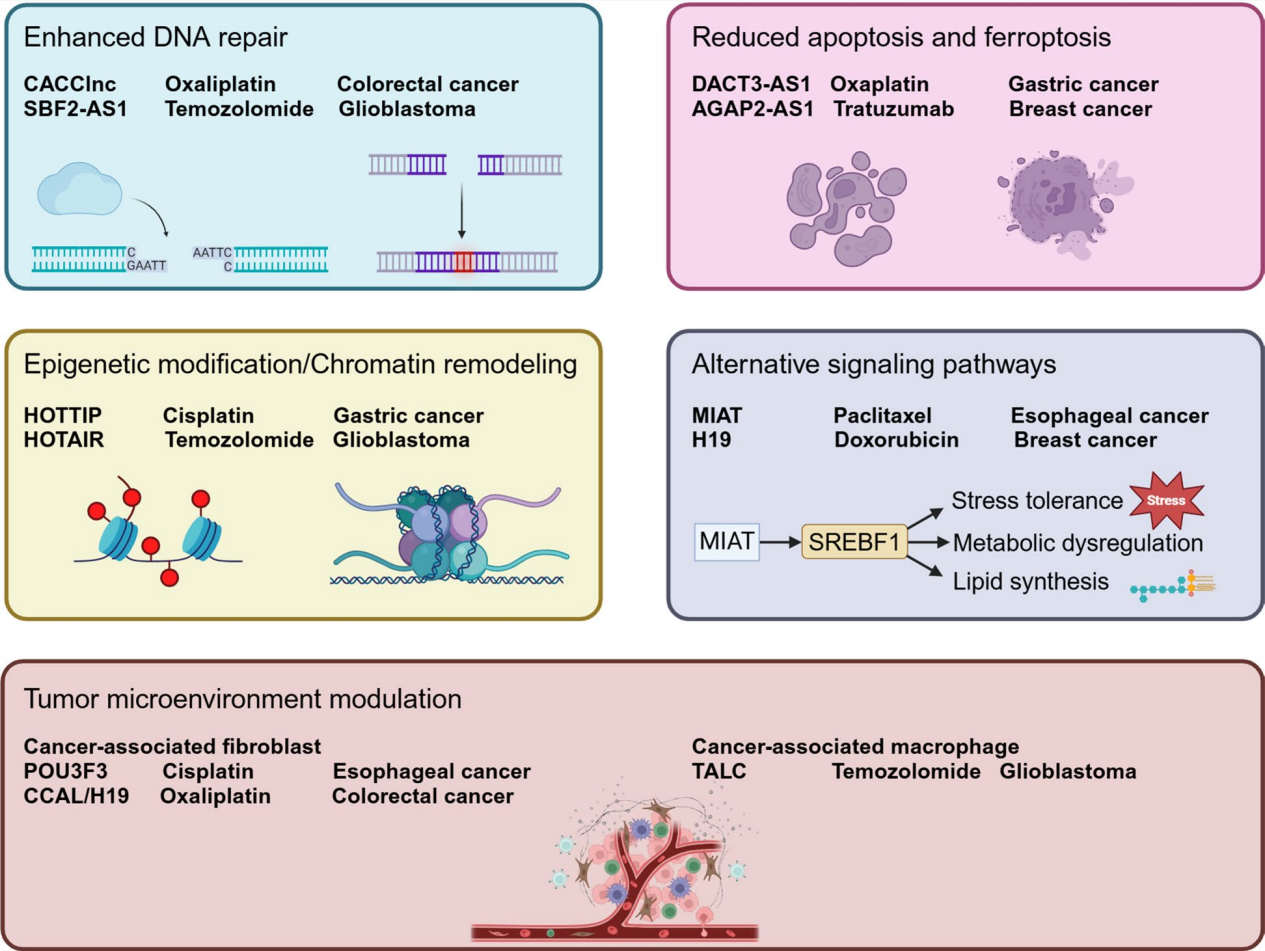
Key Mechanisms of lncRNA-Mediated Chemoresistance in Cancer. This figure highlights mechanisms by which long non-coding RNAs (lncRNAs) drive chemoresistance in cancer. First, enhanced DNA repair by lncRNAs such as CACC1nc and SBF2-AS1 increases resistance to drugs like Oxaliplatin and Temozolomide in colorectal cancer and glioblastoma. Second, reduced apoptosis and ferroptosis, facilitated by lncRNAs such as DACT3-AS1 and AGAP2-AS1, protects gastric and breast cancer cells from chemotherapy-induced cell death. Third, epigenetic modification and chromatin remodeling, mediated by lncRNAs like HOTTIP and HOTAIR, fosters resistance to Cisplatin and Temozolomide in gastric cancer and glioblastoma. Fourth, alternative signaling pathways, modulated by lncRNAs MIAT and H19, contribute to resistance in esophageal and breast cancers against Paclitaxel and Doxorubicin. Finally, tumor microenvironment modulation involving lncRNAs like POU3F3 and CCAL from cancer-associated fibroblasts, and H19 from TAMs. They are indicated to enhance resistance to Cisplatin, Oxaliplatin, and Temozolomide in esophageal, colorectal cancer, and glioblastoma, respectively. Created in BioRender

In summary, exosomal lncRNAs contribute to chemoresistance through various mechanisms, including the enhancement of DNA repair, modulation of epigenetic and chromatin remodeling pathways, alteration of the tumor microenvironment and introducing alternative signaling pathways. These insights not only underscore the complexity of chemoresistance but also provide potential therapeutic targets for overcoming this major obstacle in cancer treatment. Understanding the specific roles of exosomal lncRNAs in chemoresistance could pave the way for novel strategies aimed at sensitizing cancer cells to chemotherapy and improving patient outcomes (Fig. 3).

## Clinical significances of exosomal lncRNAs in GI cancer

### Exosomal lncRNA as diagnostic biomarkers for GI cancer

Due to the differential expression of lncRNAs across various tissues and cells, and their remarkable stability in body fluids without degradation by RNA molecules, they show significant potential as tumor diagnostic biomarkers [39] (Table 2). Compared to traditional microscopic examination and tissue biopsy, using exosomal lncRNA in liquid biopsy offers a non-invasive alternative, reflecting the real-time status of primary tumor cells, making it appealing for early diagnosis and prognosis [107]. Additionally, exosomal lncRNAs have demonstrated high diagnostic efficacy in various cancers, including gastric cancer, laryngeal squamous cell carcinoma, and colorectal cancer. Meta-analyses have shown a combined sensitivity of 0.74 and a specificity of 0.81, further supporting their potential as reliable biomarkers [108]. For instance, Cui et al. [109] utilized machine learning to identify a panel of 20 exosome-related lncRNA signatures linked to the prognosis and biomarkers of immunotherapy in ovarian cancer. This demonstrated the diagnostic and prognostic potential of exosomal lncRNAs in ovarian cancer, while also creating models for predicting prognosis and response to immunotherapy. In bladder cancer, exosomal lncRNA BCYRN1 was significantly elevated in the serum of patients compared to healthy donors, suggesting its utility as a diagnostic marker. Moreover, BCYRN1 levels dropped significantly after complete tumor resection, indicating its potential in monitoring therapeutic response [110]. Similarly, in non-small cell lung cancer, exosomal lncRNA UFC1 promotes cancer progression and is highly expressed in exosomes derived from NSCLC cells and patient serum, underscoring its role as a diagnostic and prognostic marker [111].

**Table 2:** Clinical characteristics of exosome LncRNA in GI cancer

Cancer type	Lncrna	Exosome source	Expression	Drug	Effect	Regulatory mechanisms or related moleculars	Pathway	Publication year	Ref
Colorectal cancer (CRC)	CACClnc	Tumor cells	Up	Oxaliplatin	DNA repair	Up-regulation of YB1/U2AF65 and RAD51 alternative splicing	YB1/U2AF65/RAD51 axis	2023	[43]
	FAL1	Tumor cells	Up	Oxaliplatin	Apoptosis	Up-regulation of TRIM3 and down-regulation of Beclin	Beclin1 and TRIM3	2024	[44]
	CCAL	CAFs	Up	Oxaliplatin	Apoptosis	Up-regulation of HuR and β-catenin	Wnt/β-catenin signaling	2020	[12]
	H19	CAFs	Up	Oxaliplatin	Apoptosis	Down-regulation of miR-141 and β-catenin	Wnt/β-catenin signaling	2018	[15]
	SNHG11	Tumor cells	Up	bevacizumab	Efflux pump	Down-regulation of miR-1207-5pmiR-1207-5p and ABCC1	miR-1207-5p/ABCC1 axis	2022	[14]
	PGM5-AS1	Tumor cells	Down	Oxaliplatin	Cell proliferation	Up-regulation of SRSF3 and down-regulation of PAEP; Up-regulation of miR‐423‐5p and NME1	miR‐423‐5p/NME1 axis; SRSF3/PAEP axis	2022	[45]
Esophageal cancer (EC)	MIAT	Tumor cells	Up	Paclitaxel (PTX)	Metabolism dysregulation	Up-regulation of TAF1 and SREBF1	TAF1/SREBF1 axis	2023	[46]
	POU3F3	Tumor cells	Up	Cisplatin	EMT	Differentiation of NF into CAFs is induced and IL-6 secretion is increased	IL-6 pathway	2020	[47]
Gastric cancer (GC)	MALAT1	TAMs	Up	Oxaliplatin	Enhanced glycolysis	Stabilize delta-catenin Up-regulated expression of miR-217-5p, β-catenin and HIF-1α	miR-217-5p/HIF-1α axis	2024	[48]
	DACT3-AS1	CAFs	Down	Oxaliplatin	Cell proliferation	Down-regulation of miR-181a-5p and up-regulation of sirtuin 1(SIRT1)	miR-181a-5p/SIRT1axis; KEGG pathway	2023	[49]
	HOTTIP	Tumor cells	Up	Cisplatin	EMT	Up-regulation of miR-218 and activation of HMGA1	HMGA1/miR-218 axis	2019	[50]
	CRNDE	TAMs	Up	Cisplatin	Apoptosis	Recruit NEDD4‐1 to PTEN, therefore suppresses the activation of the PI3K/Akt signaling pathway	CRNDE/PTEN axis; PI3K/Akt signaling pathway	2021	[51]
Pancreatic cancer (PC)	UCA1	PSCs	Up	Gemcitabine	Apoptosis	Up-regulation of EZH2 and down-regulation of SOCS3	SOCS3/EZH2 axis	2021	[52]
	ROR	Tumor cells	Up	Gemcitabine	Apoptosis	Down-regulation of Hippo and YAP	Hippo Signaling	2023	[53]
Hepatocellular cancer (HCC)	ROR	Tumor cells	Up	Sorafenib, doxorubicin	Cell proliferation	Up-regulation of TGFβ	TGFβ signaling pathway	2014	[54]
	H19	Tumor cells	Up	Propofol	Apoptosis	Up-regulation of miR-520a-3p and LIMK1	miR-520a-3p/LIMK1 axis	2020	[55]

Exosomal lncRNAs are emerging as promising tools for the early detection and treatment of cancer metastasis. Their involvement in regulating tumor growth, metastasis, and drug resistance highlights their importance in personalized oncology. However, several factors complicate their clinical application, such as the lack of standardized methods for exosome isolation and purification, the need for improved detection technologies with higher specificity and sensitivity, and the inherent heterogeneity of exosomal lncRNAs [112]. Despite advancements in RNA sequencing and next-generation sequencing that have enhanced the identification and analysis of exosomal lncRNAs [113], further research is needed to improve the accuracy and reliability of these methods for clinical use.

### Potential therapeutic approaches targeting exosomal lncRNAs for GI cancer treatment

Given the critical role that exosomal lncRNAs play in chemotherapy resistance, targeting these molecules offers a promising therapeutic approach to overcoming such resistance. Current strategies for targeting exosomal lncRNAs include antisense oligonucleotides (ASOs), RNA interference (RNAi), and miRNA Mimics and so on.

ASOs are short nucleic acid sequences that bind to specific RNA sequences, thereby inhibiting the expression or function of lncRNAs. They hybridize with exosomal lncRNAs, preventing their interaction with target molecules and disrupting their post-transcriptional regulatory functions [114]. This blockade can reduce the influence of lncRNAs on gene expression, potentially reversing chemotherapy resistance. ASOs have demonstrated effectiveness in inhibiting lncRNA activity in several cancer types. For instance, the lncRNA MALAT1 is associated with the development of chemotherapy resistance in renal cell carcinoma. Silencing MALAT1 has been shown to enhance chemosensitivity and suppress tumor growth [115]. In preclinical models, ASOs targeting MALAT1 effectively reduced its expression, leading to decreased tumor growth and metastasis [116], suggesting that ASOs could be a viable treatment option for MALAT1-related chemotherapy resistance.

RNAi technology, utilizing small interfering RNA (siRNA) or short hairpin RNA (shRNA), targets specific lncRNAs to promote their degradation or inhibit their translation [117]. By reducing the expression of specific lncRNAs, RNAi weakens their role in chemotherapy resistance. For example, studies have shown that targeting lncRNA PVT1 with siRNA can inhibit resistance of cancer cells to cisplatin and other drugs, providing experimental evidence that RNAi-based interference with exosomal lncRNAs can enhance chemotherapy efficacy [118].

MiRNA Mimics are another therapeutic approach that indirectly counteracts chemotherapy resistance by restoring miRNA activity. Exosomal lncRNAs often act by sequestering and inhibiting miRNAs. miRNA mimics restore or supplement miRNA function, reducing the resistance-associated activities of lncRNAs [119]. For instance, miR-34a mimics have been shown to inhibit chemotherapy resistance by disrupting the interaction between lncRNAs and their target genes [120].

The tumor microenvironment, in addition to exosomal lncRNAs derived from cancer cells, plays a crucial role in the development of chemoresistance. One such exosomal lncRNA, metastasis-associated lung adenocarcinoma transcript 1 (MALAT1), has been identified as being produced by M2 TAMs, where it promotes both glycolysis and cancer progression [121]. Given recent advancements in immunotherapy, including the use of immune cells and adjuvant therapies targeting the immune system for cancer treatment, targeting macrophages responsible for secreting this lncRNA could offer a promising approach to mitigate the development of chemoresistance and metastasis in cancer patients [122, 123].

In addition to targeting lncRNAs with various molecules to modulate their function, recent studies have developed strategies to design lncRNAs that counteract chemoresistance, delivering them to cancer cells via exosomes. For example, PGM5 antisense RNA 1 (PGM5-AS1) has been artificially delivered to colon cancer cells in combination with oxaliplatin through exosomes to mitigate resistance to the drug. Mechanistically, PGM5-AS1 recruits SRSF3, which subsequently downregulates PAEP expression, contributing to reduced chemoresistance. Furthermore, PGM5-AS1 acts as a molecular sponge for has-miR-423-5p, leading to the upregulation of NME1, which enhances the therapeutic efficacy of oxaliplatin [45].

Several targeted therapeutic strategies have shown effectiveness in inhibiting resistance-related lncRNAs. Animal models have demonstrated that lowering the levels of specific exosomal lncRNAs can restore sensitivity to chemotherapy drugs and improve tumor prognosis. Currently, several ASOs and small molecule inhibitors are undergoing clinical trials to assess their efficacy and safety in suppressing lncRNAs in cancer. However, chemotherapy resistance often involves multiple molecular pathways, and targeting a single lncRNA may not comprehensively inhibit resistance mechanisms. Therefore, combination therapies or multi-targeted approaches may be more effective and these therapeutic strategies targeting or using exosomal lncRNAs directly may show promising effect in GI cancer treatment.

### Clinical investigation of ASOs and small molecule inhibitors

Although the therapeutic mechanisms of ASOs, RNA interference, and small molecule inhibitors targeting lncRNAs have been extensively demonstrated in preclinical settings, their clinical translation remains a growing but underexplored field. Several lncRNA-targeting therapies are currently being evaluated in early-phase clinical trials, showing promise for GI cancer.

In the gastrointestinal cancer context, AZD9150 (danvatirsen), an STAT3-targeted ASO, which has undergone Phase I clinical evaluation (NCT02499328) in advanced solid tumors, including hepatocellular carcinoma [124]. Though the Phase I and Ib studies of AZD9150 in patients with advanced and metastatic HCC demonstrated limited efficacy outcomes, but the trials confirmed the feasibility of using AZD9150 as a treatment option [125].

BC-819 (also called DTA-H19) is one of the most notable small molecule inhibitors’ examples. It is a plasmid-based DNA therapy that targets the H19 lncRNA promoter, leading to the expression of diphtheria toxin A selectively in H19-expressing tumor cells [126]. BC-819 has been tested in multiple early-phase trials for bladder [127], ovarian [128], and pancreatic cancers, including a Phase IIb study (NCT01413087), which demonstrating initial safety and tumor-specific activity [129].

To date, the number of small molecule inhibitors targeting lncRNAs in clinical trials remains limited. However, ongoing high-throughput screening efforts and structural studies of lncRNAs are expected to expand the therapeutic landscape [130]. Future clinical development will benefit from integrating structural RNA biology, computational docking, and compound optimization to identify lncRNA-specific inhibitors with favorable pharmacokinetic and safety profiles.

### Potential lncRNAs-associated mechanisms leading to chemoresistance or tumor advancement in GI cancer

We have previously mentioned many lncRNAs involved in the development of chemoresistance in cancer, and each of them are associated with a different signaling pathway leading to different effects. However, many lncRNAs are also found to be associated with other key steps in cancer development and advancement but may not be associated with chemoresistance to a certain kind of drug specifically. Chemoresistance is complex and various changes in cancer can lead to the development of chemoresistance, so it is suspected that these changes induced by exosomal lncRNAs can also lead to chemoresistance or assist other chemoresistance mechanisms, and future study are expected to dig out the potential of them in chemoresistance or treatment target on them for better outcomes.

An exosomal lncRNA, FMR1-AS1, is found to facilitate the maintenance of cancer cell stemness via TLR7/NFκB/c-Myc signaling in female esophageal carcinoma [71]. And molecules involved in this signaling pathways have been demonstrated to be able to take part in the development of various cancers, including colorectal cancer, gastric cancer, pancreatic cancer, etc. [13, 131, 132]

Such complex intercalation provides promising suspicion to target these exosomal lncRNAs as adjuvant therapy for better prognosis.

By integrating current exosomal lncRNA-targeted cancer therapies and the established or potential mechanisms by which exosomal lncRNAs contribute to chemoresistance, it is strongly believed that targeting tumor-derived exosomal lncRNAs presents a promising future direction for cancer treatment. This approach could be applied either as a primary treatment strategy, preventing normal cells from undergoing malignant transformation through the transfer of exosomal lncRNAs from existing cancer cells, or as an adjuvant therapy to counteract chemoresistance in conjunction with conventional treatments. Moreover, given the successful application of specific exosomal lncRNAs, such as FMR1-AS1, in cancer therapy, this strategy holds great potential beyond oncology. Harnessing the therapeutic properties of exosomal lncRNAs could extend to other diseases rooted in genetic abnormalities or those that can be treated via genetic modification or interference. This exciting molecular tool could thus revolutionize treatment strategies for a wide array of genetic disorders.

Despite the treatment potential of exosomal lncRNAs, there remains an urgent need for further research into the biology of exosomes and the lncRNAs they transmit. By advancing our understanding of exosomal lncRNA-mediated chemoresistance, particularly in GI cancers, we may unlock novel therapeutic approaches that are more effective and capable of overcoming resistance. Continued investigation in this field could lead to the development of refined and targeted treatment strategies, significantly improving patient outcomes.

### Future perspective and conclusion

In this review, we have explored the production, secretion, transmission, and intracellular signaling of chemoresistance-related exosomal lncRNAs in GI cancers. These exosomal lncRNAs are derived from various sources, such as cancer cells, TAMs, and CAFs, contributing to chemoresistance through multiple mechanisms. By modulating genetic material and intracellular signaling, exosomal lncRNAs play a significant role in cancer progression, particularly in the development of chemoresistance.

In addition to their role in chemoresistance, exosomal lncRNAs influence other cancer-related processes, such as enhancing cancer cell stemness, promoting EMT, and inhibiting apoptosis. While not all exosomal lncRNAs involved in GI cancer have been directly linked to chemoresistance, they may contribute to other mechanisms that ultimately support the development of resistance. Further investigation into the roles of exosomes and exosomal lncRNAs could enhance our understanding of their involvement in both cancer progression and resistance to therapy.

Moreover, exosomal lncRNAs have emerged as potential therapeutic targets. Strategies such as ASOs, RNAi, and miRNA mimics have been successfully developed to target cancer-related exosomal lncRNAs. Additionally, these lncRNAs are being explored as tools to combat cancer by exploiting their interactions with DNA, RNA, and other molecules within cancer cells and the tumor microenvironment. The successful use of exosomal lncRNAs in cancer treatment highlights their potential in developing personalized therapies. By designing lncRNAs that specifically target mutations driving cancer, it may be possible to inhibit cancer cell proliferation and metastasis, or even induce cancer cell death.

**Fig. 4:**
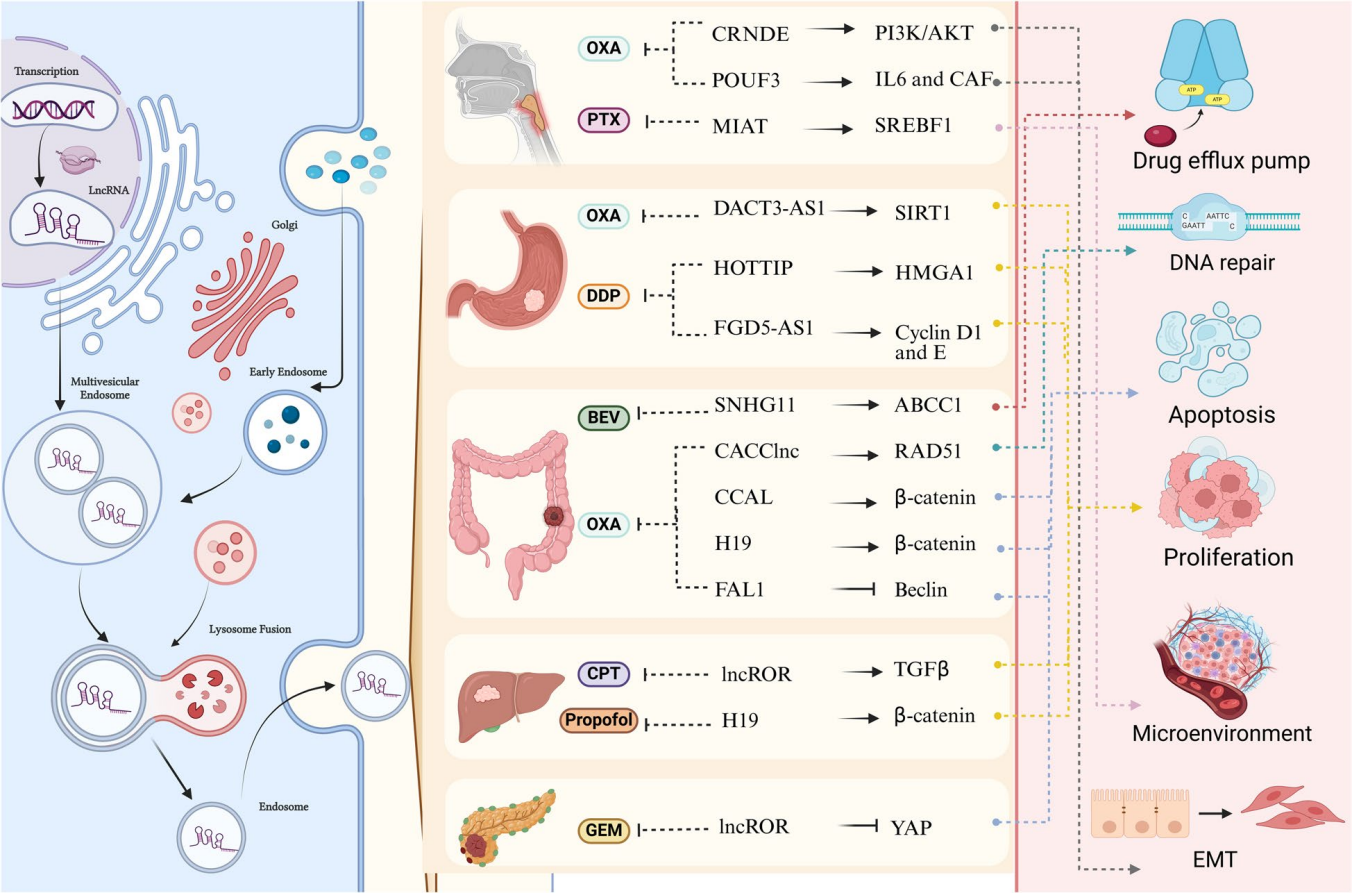
Comprehensive overview of exosomal lncRNAs and their mechanistic roles in mediating chemoresistance across gastrointestinal cancers. The left panel depicts the biogenesis and secretion of exosomal lncRNAs via the endosomal sorting pathway, including multivesicular body formation and exosome release. The middle panel details specific lncRNAs associated with resistance to various chemotherapeutic agents, including oxaliplatin (OXA), cisplatin (DDP), paclitaxel (PTX), bevacizumab (BEV), camptothecin (CPT), gemcitabine (GEM), and propofol, highlighting their involvement in key regulatory pathways such as PI3K/AKT, β-catenin signaling, and SIRT1 modulation. These lncRNAs influence critical cellular processes, including drug efflux, DNA repair, apoptosis suppression, cellular proliferation, tumor microenvironment remodeling, and epithelial-to-mesenchymal transition (EMT). The right panel summarizes the downstream functional consequences of these pathways, emphasizing their contributions to therapeutic resistance and tumor progression. Created in Biorender

In summary, exosomal lncRNAs play a pivotal role in the spread and development of chemoresistance in GI cancers through various mechanisms (Fig. 4). Concerning the diversity of chemoresistance-related exosomal lncRNAs and their complicated downstream effects, a deeper understanding of their roles could lead to novel therapeutic approaches and the development of more precise, mutation-targeted treatments. Continued research in this area holds great promise for improving chemotherapy efficacy and advancing personalized cancer therapies based on the molecular interference capabilities of exosomal lncRNAs.

## Disclaimer

None.

## Data Availability

All data supporting the conclusion of this article are included in this published article.

## Abbreviations

ABC: ATP-binding cassette
ABCC: ATP-binding cassette subfamily C member
ASOs: Antisense oligonucleotides
CACClnc: Chemoresistance-associated colorectal cancer lncRNA
CAFs: Cancer-associated fibroblasts
CRNDE: Colorectal neoplasia differentially expressed
DACT3-AS1: Disheveled binding antagonist of beta catenin 3 antisense 1
ceRNAs: Competitive endogenous RNAs
EMT: Epithelial-mesenchymal transition
ESCRT: Endosomal sorting complex required for transport
EZH2: Enhancer of zeste homolog 2
GI cancer: Gastrointestinal cancer
Hippo/YAP: Hippo/Yes-associated protein
HMGA1: High-mobility group AT-hook 1
HOTTIP: HOXA transcript at the distal tip
HuR: Human antigen R
ILVs: Intraluminal vesicles’
LIMK1: LIM domain kinase 1
lncRNAs: Long non-coding RNAs
LncROR: Long non-coding RNA regulator of reprogramming
MALAT1: Metastasis-associated lung adenocarcinoma transcript 1
MDR1: Multidrug resistance protein 1
MIAT: Myocardial infarction-associated transcript
miRNA: MicroRNA
m6A: N6-methyladenosine
NEDD4-1: Neural precursor cell expressed developmentally downregulated protein 4–1
PAEP: Progestogen-associated endometrial protein
PTEN: Phosphatase and tensin homolog
PGM5-AS1: PGM5 antisense RNA 1
RNAi: RNA interference
RRM1: Ribonucleotide reductase M1
shRNA: Short hairpin RNA
siRNA: Small interfering RNA
SNHG11: Small nucleolar RNA host gene 11
SOCS3: Suppressor of cytokine signaling 3
SREBF1: Sterol regulatory element binding transcription factor 1
SRSF3: Serine/arginine-rich splicing factor 3
TAF1: TATA-box binding protein-associated Factor 1
TALC: Temozolomide associated lncRNA in GBM recurrence
TAMs: Tumor-associated macrophages
TGFβ: Transforming growth factor-beta
TRIM3: Tripartite Motif Containing 3
UCA1: Urothelial carcinoma-associated 1
U2AF65: U2 auxiliary factor 65
VEGF: Vascular endothelial growth factor
YB1: Y-box binding protein 1
